# Microenvironment Influences on Human Umbilical Cord Mesenchymal Stem Cell-Based Bone Regeneration

**DOI:** 10.1155/2021/4465022

**Published:** 2021-08-17

**Authors:** Lingling E, Rongjian Lu, Jianwei Sun, Hongbo Li, Wen Xu, Helin Xing, Xing Wang, Tao Cheng, Shuo Zhang, Xiaocao Ma, Rong Zhang, Hongchen Liu

**Affiliations:** ^1^Institute of Stomatology & Oral Maxilla Facial Key Laboratory, First Medical Center of Chinese PLA General Hospital, Beijing 100853, China; ^2^Department of Stomatology, Fifth Medical Center of Chinese PLA General Hospital, Beijing 100071, China; ^3^Guangzhou Special Service Recuperation Center of PLA Rocket Force, Guangzhou, 510010 Guangdong Province, China; ^4^Institute for the Prevention and Control of Major Health and Public Safety Events of Armed Police, No. 9 Fuan Street, Beijing 102600, China

## Abstract

The microenvironment, or niche, regulates stem cell fate and improves differentiation efficiency. Human umbilical cord mesenchymal stem cells (hUC-MSCs) are ideal cell source for bone tissue engineering. However, the role of the microenvironments in hUC-MSC-based bone regeneration is not yet fully understood. This study is aimed at investigating the effects of the *in vitro* culture microenvironment (hUC-MSCs, nano-hydroxyapatite/collagen/poly (L-lactide) (nHAC/PLA), osteogenic media (OMD), and recombinant human bone morphogenetic protein-7 (rhBMP-7)) and the *in vivo* transplanted microenvironment (ectopic and orthotopic) on bone regeneration ability of hUC-MSCs. The isolated hUC-MSCs showed self-renewal potential and MSCs' characteristics. In the *in vitro* two-dimensional culture microenvironment, OMD or OMD with rhBMP-7 significantly enhanced hUC-MSCs' osteocalcin immunofluorescence staining, alkaline phosphatase, and Alizarin red staining; OMD with rhBMP-7 exhibited the highest ALP secretion and mineralized matrix formation. In the *in vitro* three-dimensional culture microenvironment, nHAC/PLA supported hUC-MSCs' adhesion, proliferation, and differentiation; the microenvironment containing OMD or OMD and rhBMP-7 shortened cell proliferation progression and made osteogenic differentiation progression advance; rhBMP-7 significantly attenuated the inhibiting effect of OMD on hUC-MSCs' proliferation and significantly enhanced the promoting effect of OMD on gene expression and protein secretion of osteogenic differentiation markers, calcium and phosphorous concentration, and mineralized matrix formation. The *in vitro* three-dimensional culture microenvironment containing OMD and rhBMP-7 induced hUC-MSCs to form the most new bones in ectopic or orthotopic microenvironment as proved by microcomputed tomography and hematoxylin and eosin staining, but bone formation in orthotopic microenvironment was significantly higher than that in ectopic microenvironment. The results indicated that the combination of *in vitro* hUC-MSCs+nHAC/PLA+OMD+rhBMP-7 microenvironment and *in vivo* orthotopic microenvironment provided a more optimized niche for bone regeneration of hUC-MSCs. This study elucidates that hUC-MSCs and their local microenvironment, or niche, play an important role in hUC-MSC-based bone regeneration. The endogenously produced BMP may serve an important regulatory role in the process.

## 1. Introduction

The repair of jaw bone defect has been a major challenge in surgical treatment [1]. Autologous bone grafts gain satisfactory outcome in clinical treatment with the significant limitations including insufficient bone mass, donor site morbidity, and requirement of a second surgery [2]. Stem cell-based bone tissue engineering is an alternative approach using scaffold in combination with stem cells and osteogenic factors [3]. For bone defect repair, the microenvironment within which stem cells reside, or niche, plays an important role in regulating stem cell fate and improves differentiation efficiency and has been an important factor to start-up tissue regeneration.

A typical stem cell-based bone tissue engineering requires stem cells to depart their native donor microenvironment, be cultured in the *in vitro* culture microenvironment, transplant to the *in vivo* recipient microenvironment, differentiate toward desired osteogenic lineages, and participate in bone formation. These microenvironments provide dynamic physical and chemical cues essential to their survival, proliferation, and differentiation [4]. The stem cells' donor microenvironment where they are harvested affects the regenerative ability of transplanted stem cells [5]. Bone marrow-derived mesenchymal stem cells (BM-MSCs) possessing broad characteristics of MSCs are currently regarded as the “gold standard” among MSCs [6]. However, the *in vivo* aging microenvironment results in endogenous BM-MSC dysfunction, which is also reflected in their therapeutic efficacy when used as exogenously transplanted stem cells [5]. BM-MSCs derived from aging donors showed depressed cell viability, osteogenic differentiation potential, and bone regeneration ability [7]. Human umbilical cord mesenchymal stem cells (hUC-MSCs) have been suggested as an ideal candidate of MSCs. Unlike BM-MSCs, hUC-MSCs have a clinical waste source, a painless collection procedure, and faster self-renewal properties. They can differentiate into the three germ layers that promote tissue repair and modulate immune responses and do not produce immune rejection during transplantation [8–17]. Compared with human BM-MSCs or human adipose tissue-derived MSCs, hUC-MSCs can form more calcium nodules when induced by osteogenic factor for 35 days [15]. Thus, they are attractive autologous or allogenic stem cells for the treatment of bone defect.

The regenerative ability of transplanted exogenous stem cells is also strongly influenced by the *in vitro* culture microenvironment where they are cultured. The *in vitro* culture microenvironment for stem cells is the so-called “synthetic niche.” Synthetic niches can be defined as three-dimensional culture system mimicking the interactions between stem cells and the extracellular surroundings, including biophysical factor (scaffold) and biochemical factor (growth factor) [18]. The *in vitro* three-dimensional culture system can expand the scale of stem cell culture and increase the efficiency of culture, which can better simulate the microenvironment *in vivo* when combining scaffold and growth factor to promote the directional tissue differentiation [19]. Scaffold not only provides temple for stem cells in cell adhesion, proliferation, and migration but also serves as a microenvironment for controlling tissue structure and for guiding stem cell differentiation and tissue regeneration [20]. Nanohydroxyapatite/collagen/poly (L-lactide) (nHAC/PLA), a developed ceramic/polymer composite material, mimics the nano- to microscale hierarchical microstructure of natural, cancellous bones [21], which is composed of hydroxyapatite, collagen, and polylactic acid, with a porosity of 70-88%, a pore size range of 300 ± 250 nm. Our previous and other studies have evidenced that nHAC/PLA supported stem cells' adhesion, proliferation, differentiation, and the use of in periodontal and other types of bone regeneration [22–24]. However, nHAC/PLA controls hUC-MSCs' cellular processes by building complex tissue-like structures resembling the niche to is not yet fully clear.

Growth factor is another crucial component of the *in vitro* culture microenvironment, which improves differentiation efficiency, and induces differentiation of stem cells into the desired cell lineage [19]. It is increasingly evident that stem cells undergo differentiation when cultured in the appropriate microenvironment engineered by growth factor and scaffold [25]. During normal fracture healing, bone morphogenetic proteins (BMPs) and regulatory cytokines can promote the proliferation of undifferentiated MSCs and thus induce them to differentiate into chondrocytes and osteoblasts to form bone and repair damage [26, 27]. Bone morphogenetic protein-7 (BMP-7) has been reported to promote bone healing in spinal fusion, fracture repair, and distraction osteogenesis in animals and humans [28, 29], and it has been approved by the Food and Drug Administration for adjuvant treatment of various clinical musculoskeletal diseases [30]. Early experiments demonstrated that recombinant human bone morphogenetic protein-7 (rhBMP-7) can individually induce all types of mesenchymal precursor cells into chondroblasts or osteoblasts [31, 32]. The mesoporous bioglass/silk fibrin scaffold combined with BMP-7 significantly promoted bone regeneration of osteoporotic femoral defects [33]. Study has shown that exogenously added rhBMP-7 enhances osteogenic differentiation of BM-MSCs by promoting the expression of endogenous BMP profiles [32]. Therefore, rhBMP-7 is an attractive growth factor for stem cells' osteogenic differentiation. However, the effects of the *in vitro* culture microenvironment engineered by scaffold combined with rhBMP-7 on hUC-MSCs' adhesion, proliferation, and differentiation is not yet fully understood.

Furthermore, the *in vivo* recipient microenvironment where the transplanted stem cells home also plays a pivotal role in stem cell-based bone regeneration [4]. The effects of the recipient microenvironment on stem cell function rely not only on the dynamic regulation of resident stem cells by surrounding niche but also on the interaction between transplanted stem cells and recipient microenvironment [34]. BM-MSCs from ovariectomized (OVX) mice failed to prevent bone loss when transplanted into OVX recipients, while adipose tissue-derived MSCs prevent bone loss [35]. Cai reported that fibular bone orthotopic bone defect microenvironment significantly improved engineered bone formation compared with spatium intramusculare of the hind limb ectopic microenvironment, and the scaffold degradation was accelerated as well [36]. It has been revealed that stem cells is easier to generate new bone in immunocompromised mice, but the recipient proinflammatory T cells in immunocompromised mice suppress the regenerative potential of stem cells [37]. Correspondingly, the systemic injection of T-regulatory cells could significantly promote the repair of calvarial defects by locoregionally transplanted stem cells through inhibiting recipient immune response and inflammation in immunocompromised mice [37]. These studies indicated a critical role of cell-recipient microenvironment interactions in stem cell-based bone regeneration.

As mentioned above, the microenvironments affect stem cell function, which is also reflected in their therapeutic efficacy when used as exogenously transplanted stem cells. Our previous study has proved that hUC-MSC-based engineered bones promote the repair of jaw bone defects [38]. However, the role of the microenvironments in hUC-MSC-based bone regeneration is not yet fully understood. Therefore, in this study, we hired nHAC/PLA material as scaffold, osteogenic media, and bone morphologic protein-7 as osteogenic factors to engineer the *in vitro* culture microenvironment and combined with the *in vivo* ectopic and orthotopic transplanted microenvironment (subcutaneous ectopic site in severe combined immunodeficient (SCID) mice and jaw bone orthotopic site in New Zealand white rabbits) to explore the effects of various ecological niches on osteogenic differentiation and bone regeneration ability of hUC-MSCs. This study elucidates that hUC-MSCs and their local microenvironment, or niche, play an important role in hUC-MSC-based bone regeneration.

## 2. Materials and Methods

### 2.1. Isolation and Culture of HUC-MSCs

An explant culture method [39] was used to culture hUC-MSCs. All surgical procedures and care administered to human samples were approved by the Medical Ethics Committee of Chinese People's Liberation Army (PLA) General Hospital (ethics approval no. S2018-093-01). Briefly, after informed consent in writing was obtained, 10 umbilical cord tissues from 10 healthy individuals with full-term pregnancy (age, 25-32 years) were collected and minced into small sections. All samples were obtained from the Maternity Department of Chinese PLA General Hospital (Beijing, China). Following the removal of the vascular, perivascular, and epithelial tissues of every small section, the remaining Wharton's Jelly was minced into 1 cm^3^ fragments. The fragments were then attached to the bottom of a culture dish and incubated at 37°C in 5% CO_2_, from which the hUC-MSCs migrated in the human MSC serum-free medium containing 500 ml human MSC serum-free basal medium (catalog no. CM-SC01, Procell Life Science Technology Co., Ltd.), 25 ml human MSC serum-free medium growth additives (catalog no. CM-SC01, Procell Life Science technology Co., Ltd.), and 5 ml gentamicin-streptomycin solution (catalog no. CM-SC01, Procell Life Science technology Co., Ltd.). The third passage cells were used to perform a series of experiments.

### 2.2. Proliferative Potential of HUC-MSCs

The hUC-MSCs were plated into 96-well culture plates at a density of 2 × 10^4^ cells/ml and then were cultured in 100 *μ*l human MSC serum-free medium for 1-10 days to test their proliferative potential using a Cell Counting Kit-8 (CCK-8) according to the manufacturer's protocol (catalog no. 35002, Dojindo Molecular Technologies, Inc.).

### 2.3. Phenotype of HUC-MSCs

Flow cytometry was used to analyze the phenotype of hUC-MSCs. Trypsinized cells were suspended in phosphate-buffered saline (catalog no. PBS-10001, Cyagen Biosciences, Inc.) at a density of 5 × 10^6^ cells/ml, and a 100 *μ*l sample was incubated with various BD Pharmingen™ PE mouse anti-human CD73 (catalog no. 561014, 1 : 50), CD105 (catalog no. 560839, 1 : 50), CD34 (catalog no. 560941, 1 : 50), and BD Pharmingen™ FITC mouse anti-human CD90 (catalog no. 561969, 1 : 50), CD45 (catalog no. 560976, 1 : 50), CD11a (catalog no. 555383, 1 : 50), and HLA-DR (catalog no. 560944, 1 : 50) antibodies (BD Biosciences) for 45 min at room temperature. Control samples were incubated with PBS instead of antibodies. Antibody binding was examined using a FACScan flow cytometer (Beckman Coulter) and was analyzed using FlowJo v10.6.2 (BD Biosciences).

### 2.4. Multilineage Differentiation Potential of HUC-MSCs

Osteogenic differentiation was induced in hUC-MSCs by Oricell™ hUC-MSCs osteogenic differentiation medium kit (catalog no. HUXUC-90021, Cyagen Biosciences, Inc.). The cells were seeded in 24-well culture plates at a density of 1 × 10^4^ cells/cm^2^ and cultured in the human MSC serum-free medium. When confluence recached 80-90%, the cells were cultured in hUC-MSC osteogenic differentiation medium for 21 days and then were fixed with 4% neutral formaldehyde for 30 min at room temperature. The extracellular matrix calcification was examined using Alizarin red staining for 5 min at room temperature. The stained cells were photographed under an inverted light microscope.

Adipogenic differentiation was induced in hUC-MSCs by Oricell™ hUC-MSC adipogenic differentiation medium kit (catalog no. HUXUC-90031, Cyagen Biosciences Inc, USA). The cells were plated onto chamber slides in 6-well plates at a density of 2 × 10^4^ cells/cm^2^ and cultured in the human MSC serum-free medium. When confluence reached 100%, the cells were cultured in hUC-MSC adipogenic differentiation medium for 21 days and then were fixed with 4% neutral formaldehyde for 30 min at room temperature and examined with Oil red O staining for 30 min at room temperature. The stained cells were photographed under an inverted light microscope.

### 2.5. Osteogenic Differentiation Ability of HUC-MSCs in the In Vitro Two-Dimensional Culture Microenvironment

The hUC-MSCs were seeded onto chamber slides in 24-well culture plates or in 24-well culture plates at a density of 1 × 10^5^ cells/cm^2^ and were cultured in the human MSC serum-free medium. When confluence reached 80-90%, the cells were cultured in 1 ml human MSC serum-free medium, 1 ml human MSC serum-free OMD, or 1 ml human MSC serum-free OMD supplemented with 100 ng/ml rhBMP-7 (catalog no. 354-BP-010/CF, R&D Systems, Inc.) [32]. The serum-free Oricell™ hUC-MSC osteogenic differentiation medium served as OMD. On day 14 of differentiation, the cells seeded onto chamber slides were fixed with 4% neutral formaldehyde for 30 min at room temperature. Immunofluorescence staining was used to examine the expression of osteocalcin (OCN). Simply, the cells were incubated with mouse anti-human OCN monoclonal antibody (catalog no. MAB1419, 1 : 50, R&D Systems, Inc.) overnight at 4°C. FITC-conjugated anti-rabbit IgG secondary antibody (1 : 50, Santa Cruz Biotechnology, Inc.) was applied for 2 h at room temperature. The nucleus was stained with 4′,6-diamidino-2-phenylindole (Merck KGaA) for 15 min at room temperature. Subsequently, the confocal images were recorded using a confocal microscope.

After the cells seeded in 24-well culture plates were cultured for 14 days, the cell culture supernatant were collected from the wells to measure alkaline phosphatase (ALP) activity (catalog no. 03333701190, alkaline phosphatase acc. to IFCC Gen.2 kit, Roche Diagnostics GmbH) using an automatic biochemical analyzer (Roche COBAS8000, Roche Diagnostics GmbH) in the Biochemistry Department of Chinese PLA General Hospital. The cells were then fixed with 4% neutral formaldehyde for 30 min at room temperature. The Gomori calcium-cobalt method [24] was then used to estimate alkaline phosphatase (ALP) activity. The mineralized matrix formation was examined using Alizarin red staining for 5 min at room temperature. The stained cells were photographed under an inverted light microscope. For mineralized matrix formation measurements, each well was eluted for 30 min at room temperature with 1 ml 10% acetic acid solution (the volume ratio of acetic acid and anhydrous ethanol is 8 : 2) on the rocking bed [40]. The absorbance values of the eluents were measured at 490 nm using a microplate reader.

### 2.6. Preparation and Seeding of NHAC/PLA Scaffolds

The nHAC/PLA materials (Beijing Allgens Medical Science & Technology Co., Ltd.) were constructed into blocks of 3.5 × 3.5 × 3.5, 5 × 5 × 5, and 10 × 4 × 3 mm. The samples were rinsed with 100% alcohol and sterilized with cobalt 60. The hUC-MSCs were seeded onto nHAC/PLA in 24-well plates and cultured in the human MSC serum-free medium for 24 h at 37°C, allowing the cells to adhere to nHAC/PLA. The medium was then changed to additional human MSC serum-free medium, human MSC serum-free OMD, or human MSC serum-free OMD supplemented with 100 ng/ml rhBMP-7. The constructs were then ready for a series of experiments.

### 2.7. Scanning Electron Microscopy

The hUC-MSCs were seeded onto 10 × 4 × 3 mm nHAC/PLA scaffolds in 24-well plates at a density of 1 × 10^7^ cells/cm^2^ per sample and were cultured in 1 ml mentioned above medium for 7 days. The constructs were fixed with 2% paraformaldehyde and 2.5% glutaraldehyde (Merck KGaA) in 0.1 mol/l phosphate buffer for 48 h at room temperature and were then rinsed with PBS, different concentrations of ethanol, and different concentrations of hexamethyldisilazane. The construct were glued with conducing paste (catalog no. C680548, 8 mm × 20 m, Nissin EM Co., Ltd.) to appropriate mounting stabs and coated with a several nanometer-thick layer of gold and examined under a Hitachi S-520 scanning electron microscope.

### 2.8. Proliferation Ability of HUC-MSCs in the In Vitro Three-Dimensional Culture Microenvironment

The hUC-MSCs were seeded onto 3.5 × 3.5 × 3.5 mm nHAC/PLA scaffolds in 96-well plates at a density of 2 × 10^4^ cells/cm^2^ per sample and were cultured in 100 *μ*l mentioned above medium for 1, 3, 5, 7, and 9 days to measure the proliferation of hUC-MSCs using CCK-8 according to the manufacturer's protocol (catalog no. 35002, Dojindo Molecular Technologies, Inc.).

### 2.9. Osteogenic Differentiation Ability of HUC-MSCs in the In Vitro Three-Dimensional Culture Microenvironment

The hUC-MSCs were seeded onto 5 × 5 × 5 mm nHAC/PLA scaffolds in 24-well plates at a density of 1 × 10^6^ cells/cm^2^ per sample and were cultured in 1.5 ml mentioned above medium for 7 and 14 days. After the cell culture supernatants were collected, the total cellular RNA was then extracted from the constructs with TRIzol reagent (catalog no. 15596-018, Thermo Fisher Scientific, Inc.) and reverse-transcribed into cDNA using a reverse transcription kit (catalog no. A5001, Promega Corporation) with annealing at 25°C for 5 min, extension at 42°C for 60 min and inactivating at 70°C for 15 min. SYBR® green real-time polymerase chain reaction (RT-PCR) master mix (catalog no. QPK-201, Toyobo Life Science) was used to quantify the target genes, including ALP, OCN, bone morphologic protein 2 (BMP-2), and GAPDH. Simply, the components of the PCR system were added and uniformly mixed to 20 *μ*l, with 95°C of predenaturation for 5 min, followed by qPCR, with denaturation at 95°C for 10 sec, annealing at 60°C for 30 sec, and extension at 72°C for 30 sec. A total of 40 cycles were performed. The 2^−ΔΔ*Cq*^ method was used to evaluate relative gene expression normalized by the *C*_*q*_ of the housekeeping gene GAPDH. The *C*_*q*_ value of hU-CMSCs+nHAC/PLA cultured in serum-free medium for 7 days served as the calibrator (biological replicates, *n* = 3; technical replicates, *n* = 3). The primer sequences used were as follows: ALP forward sequence, 5′-CTATCCTGGCTCCGTGCTC-3′, and reverse sequence, 5′-GCTGGCAGTGGTCAGATGTT-3′; and OCN forward sequence, 5′-CTCACACTCCTCGCCCTATT-3′, and reverse sequence, 5′-TTGGACACAAAGGCTGCAC-3′; and BMP-2 forward sequence, 5′-ACCCGCTGTCTTCTAGCGT-3′, and reverse sequence, 5′-TTTCAGGCCGAACATGCTGAG-3′; and GAPDH forward sequence, 5′-TCAAGAAGGTGGTGAAGCAGG-3′, and reverse sequence, 5′-GCGTCAAAGGTGGAGGAGTG-3′.

The collected cell culture supernatants were used to measure ALP activity (catalog no. 03333701190, Alkaline Phosphatase acc. to IFCC Gen.2 kit, Roche Diagnostics GmbH), OCN concentration (catalog no. 12149133122, Elecsys N-MID Osteocalcin kit, Roche Diagnostics GmbH), calcium (Ca) concentration (catalog no. 05168449190, Calcium Gen.2 (Ca 2) kit, Roche Diagnostics GmbH), and phosphorous (P) concentration (catalog no. 05171377190, Phosphate (Inorganic) ver.2 (PHOS2) kit, Roche Diagnostics GmbH) using an automatic biochemical analyzer (Roche COBAS8000, Roche Diagnostics GmbH) in the Biochemistry Department of Chinese PLA General Hospital. BMP-2 concentration in the cell culture supernatant was checked by enzyme-linked immunosorbent assay (ELISA) according to the manufacturer's protocol (catalog no. ab119581, Human BMP2 ELISA Kit, Abcam, Cambridge, MA, USA).

The constructs were cultured in human MSC serum-free OMD supplemented with 100 ng/ml rhBMP-7 microenvironment for 14 days; hU-CMSCs seeded on nHAC/PLA scaffolds were subjected to karyotyping analysis, followed by treatment with 100 *μ*g/ml colcemid (Sigma) and harvesting with 2.5% trypsin. The cells were then collected by centrifugation and stained by Giemsa staining. Karyotyping analysis was performed using G-banding techniques in the clinical laboratory of the PLA General Hospital.

The constructs were stained using Alizarin red solution for 5 min at room temperature and photographed. For mineralized matrix formation measurements, each construct was eluted for 30 min at room temperature with 1 ml 10% acetic acid solution (the volume ratio of acetic acid and anhydrous ethanol is 8 : 2) on the rocking bed. The absorbance values of the eluents were measured at 490 nm using a microplate reader. The nHAC/PLA without cells cultured in the human MSC serum-free medium was used as a blank control.

### 2.10. Ectopic and Orthotopic Surgical Procedure

**Figure 1:**
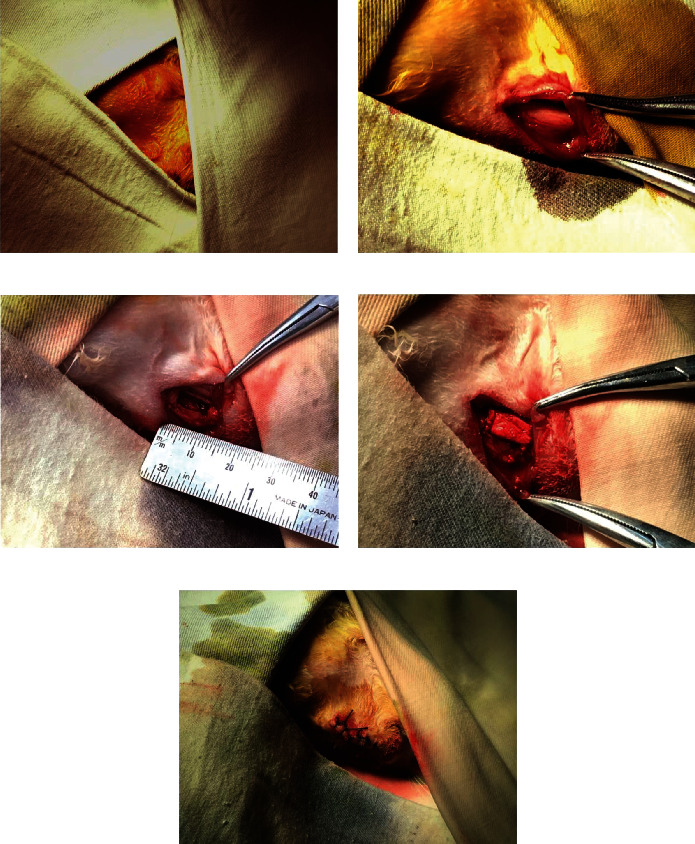
Surgical procedure. (a) Iodophor disinfection operation area and paved sterile disinfection outside the operation area. (b) Exposed rabbit mandible frontiers. (c) A 10 × 4 × 3 mm jaw bone defect. (d) Implantation of the hUC-MSCs+nHAC/PLA construct. (e) Suture incision.

The hUC-MSCs were seeded onto 10 × 4 × 3 mm nHAC/PLA scaffolds in 24-well plates at a density of 1 × 10^7^ cells/cm^2^ per sample and were cultured in 1 ml mentioned above medium for 7 days. Next, the segmental jaw bone defects (10 × 4 × 3 mm) were performed in 24 female New Zealand white rabbits (weight range of 2.50-3.00 kg, Medical Laboratory Animal Center of Chinese PLA General Hospital, Figure 1). New Zealand white rabbits in each group were administered using 0.5 ml/kg 1 : 1 (*V*/*V*) xylazine hydrochloride injection (catalog no. (2015)070011777, 0.25 mg/kg, HuaMu Animal Health Products Co., Ltd., Jilin, China) and midazolam injection (catalog no. H10980025, 5 mg/kg, Jiangsu Nhwa Pharmaceutical Co., Ltd., China) by intramuscular injection. The ectopic bone formations were performed subcutaneously on the back of 6 female severe combined immunodeficient (SCID) mice (weight range of 18-20 g, Medical Laboratory Animal Center of Chinese PLA General Hospital). The SCID mice in each group were anesthetized with pentobarbital sodium (50-60 mg/kg). And then, the nHAC/PLA, hUC-MSCs+nHAC/PLA, hUC-MSCs+nHAC/PLA+OMD, and hUC-MSCs+nHAC/PLA+OMD+rhBMP-7 were implanted into the jaw bone defects of 24 New Zealand white rabbits (1 sample per rabbit) and subcutaneously on the backs of 6 SCID mice (4 samples per mouse). New Zealand white rabbits were housed in cages in a normal environment with a temperature of 16-26°C, a relative humidity of 40-70%, and a minimum air change of 8 times per h. The SCID mice were housed in cages in a barrier environment with a temperature of 20-26°C, a relative humidity of 40-70%, and a minimum air change of 15 times per h. They were exposed to 12 h of light and 12 h of darkness every day with a regular diet and drinking water. All surgical procedures and care administered to the animals were approved by the Animal Care and Use Committee of Chinese People's Liberation Army General Hospital and were performed according to institutional guidelines (ethics approval no. 2018-X14-87).

### 2.11. Assessment of Ectopic and Orthotopic Bone Formation

After 3 months of implantation, the rabbits were sacrificed by overdose of anesthesia; the SCID mice were anesthetized and then sacrificed by cervical dislocation. The implants of jaw bone defect and the back of SCID mice were removed surgically and then were fixed in 10% formalin for 72 hours at room temperature. The implants of jaw bone defect were evaluated by microcomputed tomography (micro-CT) using the quantum GX *μ*CT system with a source voltage of 70 kV, current of 114 *μ*A, and 4.5 *μ*m accuracy. Three-dimensional images of the defects were reconstructed from the scans by the Quantum GX *μ*CT Workstation.

Next, all implants were embedded; specimens were trimmed using waterproof polishing paper without demineralization, cut into 5 *μ*m sections, and stained using hematoxylin and eosin for 5 min at room temperature. The stained sections were photographed under an inverted light microscope. For morphometric analysis, the extent of newly formed bone was indicated by the percentage of bone formation area within the section. One section was selected every 5 sections, and then, five consecutive sections per implant were obtained to evaluate the percentage of bone formation area. Five fields of view were selected for each section per implant under an inverted light microscope and were calculated using a Leica Qwin v3.2 image analysis system (Leica Microsystems Inc.). Total scores per section were calculated and averaged for all sections to obtain an overall score for each implant. Data were then averaged across 6 implants within each group.

### 2.12. Statistical Analysis

All experiments were repeated at least three independent times. Data were analyzed using SPSS 13.0 (SPSS, Inc.) and presented as the mean ± standard deviation (SD). Comparisons between two groups were assessed by Student's *t*-test, whereas multigroup comparisons were analyzed by one-way ANOVA followed by Tukey's multiple comparison test. Tamhane's T2 multiple comparison test was used on data with a nonnormal distribution or unequal variance. A *P* value of <0.05 was considered statistically significant.

## 3. Results

### 3.1. Isolation, Culture, and Identification of HUC-MSCs

**Figure 2:**
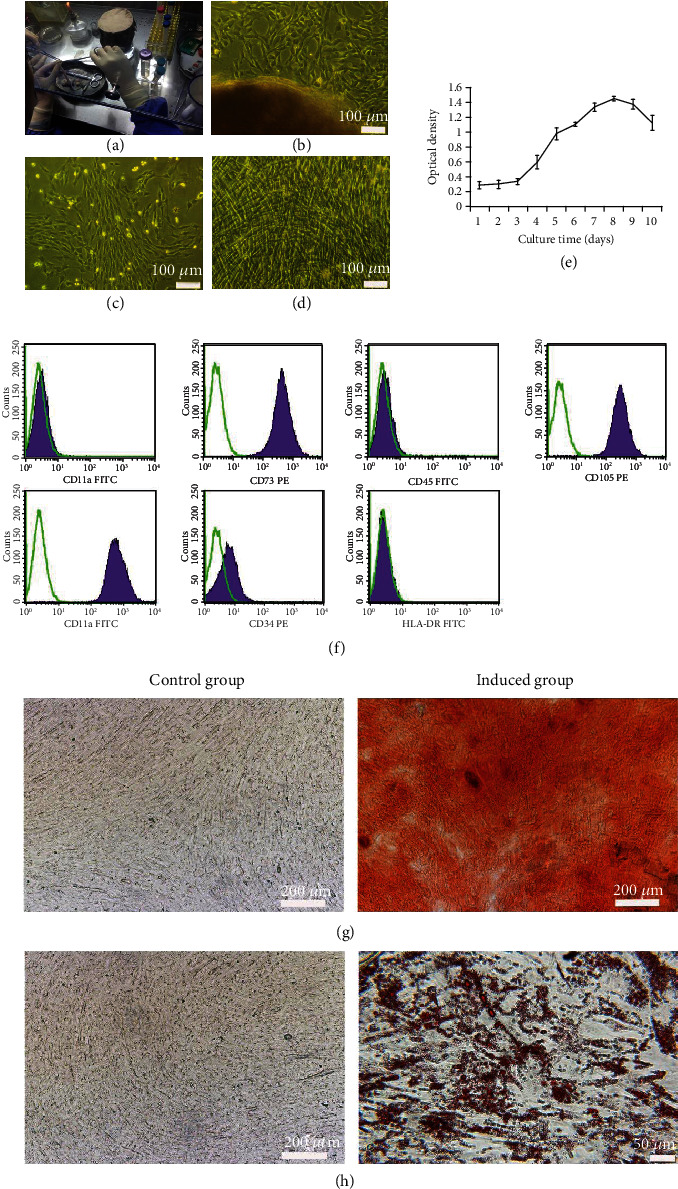
Characteristics of hUC-MSCs. (a) Umbilical cord. (b) Tissue explant culture of hUC-MSCs (scale bar = 100 *μ*m). (c) Phase 3 hUC-MSCs on day 1 of culture (scale bar = 100 *μ*m). (d) Phase 3 hUC-MSCs on day 4 of culture (scale bar = 100 *μ*m). (e) Proliferation of hUC-MSCs was evaluated using a CCK-8 assay (mean ± SD, *n* = 12). (f) Phenotype of hUC-MSCs. Green line indicates isotype control; purple-shaded histograms indicate positive staining. (g) Alizarin red staining on day 21 of culture (scale bar = 200 *μ*m). (h) Oil red O staining on day 21 of culture (control group scale bar = 200 *μ*m, induced group scale bar = 50 *μ*m).

The Wharton's Jelly was isolated from umbilical cord tissues (Figure 2(a)). The cells migrated from fragments of Wharton's Jelly after 3-4 days of culture (Figure 2(b)). The phase 3 hUC-MSCs exhibited typical fibroblastic morphology on day 1 of culture (Figure 2(c)) and were arranged in a radial or whirlpool arrangement on day 4 of culture (Figure 2(d)). In the first 3 days of the incubation period, the cells did not markedly proliferate, and they entered the logarithmic growth period on days 4-7. Cell proliferation reached the highest point on day 8 and entered the platform stage (Figure 2(e)). The cells expressed CD73 (99.78%), CD90 (99.89%), and CD105 (99.72%) and did not express CD11a (0.08%), CD45 (0.32%), CD34 (2.31%), and human leukocyte antigen HLA-DR (0.09%; Figure 2(f)). Under specific culture conditions, the cells differentiated into osteogenic (Figure 2(g)) and adipogenic (Figure 2(h)) lineages.

### 3.2. Osteogenic Differentiation Ability of HUC-MSCs in the In Vitro Two-Dimensional Culture Microenvironment

**Figure 3:**
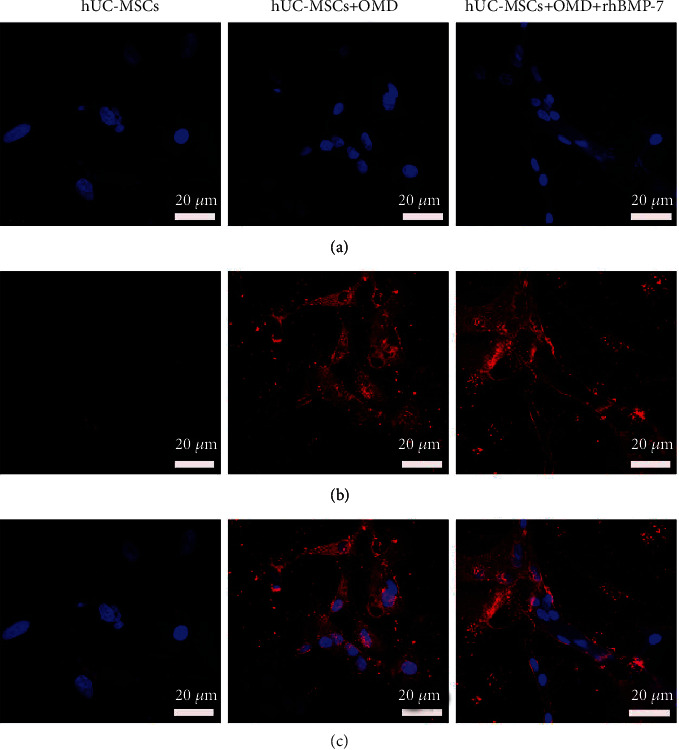
Immunofluorescence staining of OCN in hUC-MSCs in the *in vitro* two-dimensional culture microenvironment for 14 days. (a) DAPI. (b) OCN. (c) Merge. Scale bar = 20 *μ*m.

**Figure 4:**
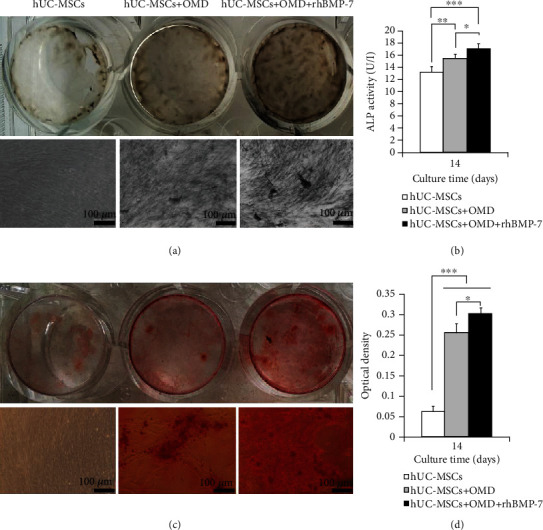
Osteogenic differentiation ability of hUC-MSCs in the *in vitro* two-dimensional culture microenvironment (mean ± SD, *n* = 6). (a) ALP staining. (b) ALP secretion. (c) Alizarin red staining. (d) Mineralized matrix formation. Scale bar = 100 *μ*m. ^∗^*P* < 0.05, ^∗∗^*P* < 0.01, ^∗∗∗^*P* < 0.001.

When cultured in the two-dimensional microenvironment containing OMD or OMD+rhBMP-7 for 14 days, the hUC-MSCs were positively stained for OCN (Figure 3) and ALP (Figure 4(a)), and the mineralized matrix were produced (Figure 4(c)). The exogenously added rhBMP-7 showed more intensive ALP staining and Alizarin red staining. Furthermore, OMD or OMD+rhBMP-7 significantly promoted ALP secretion (*P* < 0.01 and *P* < 0.001) and mineralized matrix formation (*P* < 0.001 and *P* < 0.001) of hUC-MSCs. The highest ALP secretion (*P* < 0.05) and mineralized matrix formation (*P* < 0.05) were presented in the hUC-MSCs+OMD+rhBMP-7 microenvironment (Figures 4(b) and 4(d)).

### 3.3. Proliferation and Osteogenic Differentiation Ability of HUC-MSCs in the In Vitro Three-Dimensional Culture Microenvironment

**Figure 5:**
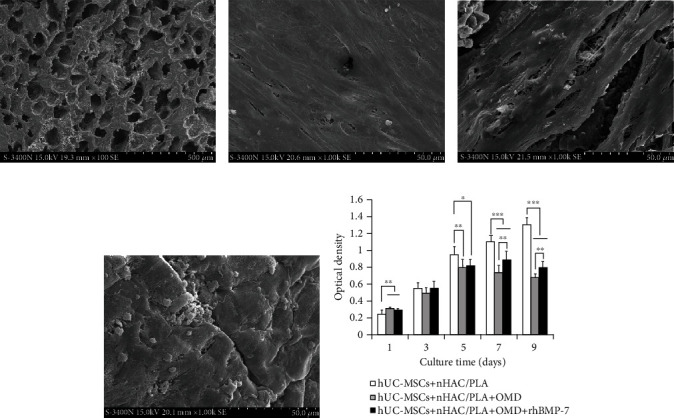
Scanning electron microscopy observation for 7 days and the proliferation ability of hUC-MSCs for 1, 3, 5, 7, and 9 days in the *in vitro* three-dimensional culture microenvironment. (a) nHAC/PLA (scale bar = 500 *μ*m). (b) hUC-MSCs+nHAC/PLA (scale bar = 50 *μ*m). (c) hUC-MSCs+nHAC/PLA+OMD (scale bar = 50 *μ*m). (d) hUC-MSCs+nHAC/PLA+OMD+rhBMP-7 (scale bar = 50 *μ*m). (e) Proliferation (mean ± SD, *n* = 8). ^∗^*P* < 0.05, ^∗∗^*P* < 0.01, ^∗∗∗^*P* < 0.001.

Scanning electron microscopy observation showed that nHAC/PLA exhibited a nano-to-microscale hierarchical architecture of natural, cancellous bones (Figure 5(a)) and provided a microenvironment for hUC-MSCs' growth. When cultured for 7 days, the uninduced cells adhered, extended, and proliferated on the surface and in the pore of the nHAC/PLA, and produced some filarious extracellular matrixes (Figure 5(b)). While induced by the microenvironment containing OMD (Figure 5(c)) or OMD+rhBMP-7 (Figure 5(d)), the cells were covered by abundant amounts of matrix protein deposits.

The influence of the *in vitro* three-dimensional culture microenvironment on cell proliferation was evaluated by CCK-8 assay. The results showed that, when the cells were cultured in the hUC-MSCs+nHAC/PLA, hUC-MSCs+nHAC/PLA+OMD, and hUC-MSCs+nHAC/PLA+OMD+rhBMP-7 microenvironments, the cell proliferation reached the highest points on days 9, 5, and 7, respectively. The microenvironments containing OMD and OMD+rhBMP-7 significantly promoted cell proliferation on day 1 (*P* < 0.01 and *P* < 0.01) and significantly suppressed cell proliferation on days 5 (*P* < 0.01 and *P* < 0.05), 7 (*P* < 0.001 and *P* < 0.001), and 9 (*P* < 0.001 and *P* < 0.001). But the hUC-MSCs+nHAC/PLA+OMD+rhBMP-7 microenvironment exhibited significantly higher cell proliferation than the hUC-MSCs+nHAC/PLA+OMD microenvironment on days 7 (*P* < 0.01) and 9 (*P* < 0.01, Figure 5(e)).

**Figure 6:**
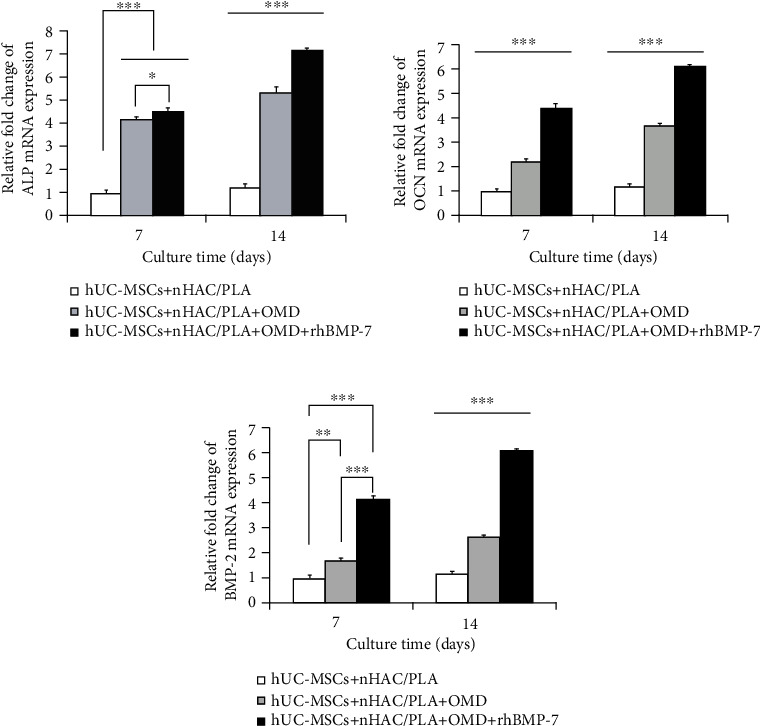
Gene expression related to osteogenic differentiation in hUC-MSCs in the *in vitro* three-dimensional culture microenvironment (mean ± SD, *n* = 3). (a) ALP. (b) OCN. (c) BMP-2. ^∗^*P* < 0.05, ^∗∗^*P* < 0.01, ^∗∗∗^*P* < 0.001.

RT-PCR was used to test the mRNA expression of osteogenic-associated genes in hUC-MSCs. The results showed that the microenvironments containing OMD and OMD+rhBMP-7 significantly upregulated ALP (*P* < 0.001), OCN (*P* < 0.001), and BMP-2 (*P* < 0.01) mRNA expressions in hUC-MSCs on days 7 and 14. And the hUC-MSCs+nHAC/PLA+OMD+rhBMP-7 microenvironment exhibited significantly higher ALP (*P* < 0.05), OCN (*P* < 0.001), and BMP-2 (*P* < 0.001) mRNA expressions than the hUC-MSCs+nHAC/PLA+OMD microenvironment (Figures 6(a)–6(c)).

**Figure 7:**
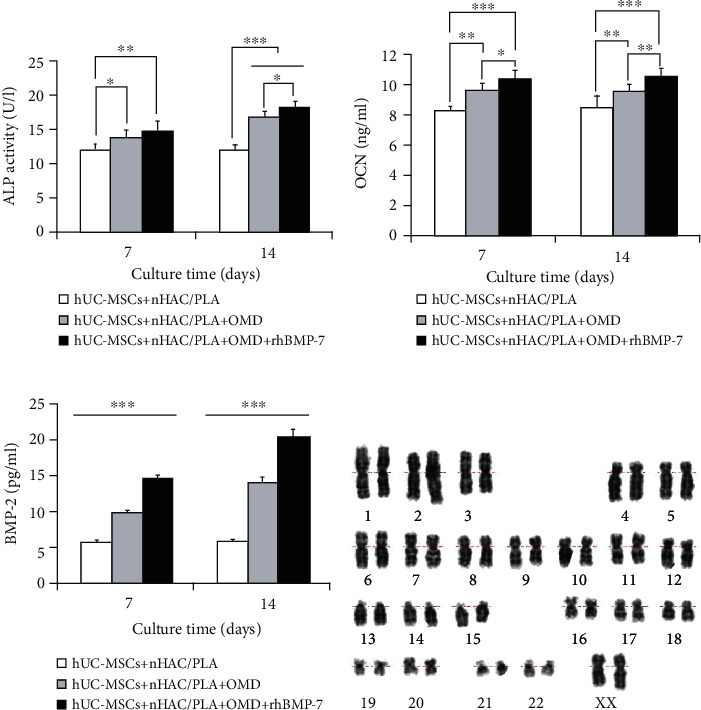
Protein secretion related to osteogenic differentiation in hUC-MSCs in the *in vitro* three-dimensional culture microenvironment (mean ± SD, *n* = 6). (a) ALP. (b) OCN. (c) BMP-2. ^∗^*P* < 0.05, ^∗∗^*P* < 0.01, ^∗∗∗^*P* < 0.001. (d) The hUC-MSCs at passage 3 that were cultured on nHAC/PLA in human MSC serum-free OMD supplemented with 100 ng/ml rhBMP-7 microenvironment exhibited a normal karyotype.

And then, the protein secretion of these osteogenic-associated genes in hUC-MSCs was examined by Roche and ELISA Kits. The results showed that the microenvironments containing OMD and OMD+rhBMP-7 significantly enhanced ALP (*P* < 0.05), OCN (*P* < 0.01), and BMP-2 secretions (*P* < 0.001) of hUC-MSCs on days 7 and 14. On day 7, the hUC-MSCs+nHAC/PLA+OMD+rhBMP-7 microenvironment exhibited significantly higher OCN (*P* < 0.05) and BMP-2 secretions (*P* < 0.001) than the hUC-MSCs+nHAC/PLA+OMD microenvironment. On day 14, the hUC-MSCs+nHAC/PLA+OMD+rhBMP-7 microenvironment exhibited significantly higher ALP (*P* < 0.05), OCN (*P* < 0.01), and BMP-2 secretion (*P* < 0.001) than the hUC-MSCs+nHAC/PLA+OMD microenvironment (Figures 7(a)–7(c)).

The constructs were cultured in human MSC serum-free OMD supplemented with 100 ng/ml rhBMP-7 microenvironment for 14 days; hU-CMSCs seeded on nHAC/PLA scaffolds exhibited a normal diploid karyotype. Chromosome structural abnormalities such as inversion, deletion, translocation, and rings were not observed by karyotyping analysis of G-banding (Figure 7(d)).

**Figure 8:**
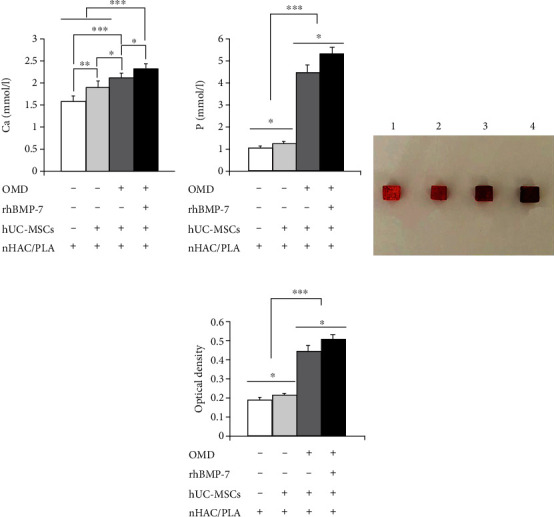
Ca concentration, P concentration, and mineralized matrix formation in the *in vitro* three-dimensional culture microenvironment for 14 days (mean ± SD, *n* = 6). (a) Ca. (b) P. (c) Alizarin Red staining. (c-1) nHAC/PLA. (c-2) hUC-MSCs+nHAC/PLA. (c-3) hUC-MSCs+nHAC/PLA+OMD. (c-4) hUC-MSCs+nHAC/PLA+OMD+rhBMP-7. (d) Mineralized matrix formation. ^∗^*P* < 0.05, ^∗∗^*P* < 0.01, ^∗∗∗^*P* < 0.001.

Furthermore, on day 14, we examined calcium and phosphorus concentrations in these microenvironments by Roche Kits. The results showed that the hUC-MSCs+nHAC/PLA, hUC-MSCs+nHAC/PLA+OMD, and hUC-MSCs+nHAC/PLA+OMD+rhBMP-7 microenvironments produced significantly higher Ca concentration (*P* < 0.05) and P concentration (*P* < 0.05) than the nHAC/PLA alone. The hUC-MSCs+nHAC/PLA+OMD and hUC-MSCs+nHAC/PLA+OMD+rhBMP-7 microenvironments produced significantly higher Ca concentration (*P* < 0.05) and P concentration (*P* < 0.001) than the hUC-MSCs+nHAC/PLA microenvironment. The highest Ca concentration (*P* < 0.05) and P concentration (*P* < 0.05) were presented in the hUC-MSCs+nHAC/PLA+OMD+rhBMP-7 microenvironment (Figures 8(a) and 8(b)).

The constructs were then stained using Alizarin red solution and photographed (Figure 8(c)). The quantitative analysis showed that the hUC-MSCs+nHAC/PLA, hUC-MSCs+nHAC/PLA+OMD, and hUC-MSCs+nHAC/PLA+OMD+rhBMP-7 constructs exhibited significantly higher mineralized matrix formation (*P* < 0.05) than the nHAC/PLA alone. The hUC-MSCs+nHAC/PLA+OMD and hUC-MSCs+nHAC/PLA+OMD+rhBMP-7 constructs exhibited significantly higher mineralized matrix formation (*P* < 0.001) than the hUC-MSCs+nHAC/PLA. The highest mineralized matrix formation were presented in the hUC-MSCs+nHAC/PLA+OMD+rhBMP-7 construct (*P* < 0.05, Figure 8(d)).

### 3.4. Engineered Bone Formation in the In Vivo Ectopic and Orthotopic Microenvironments

**Figure 9:**
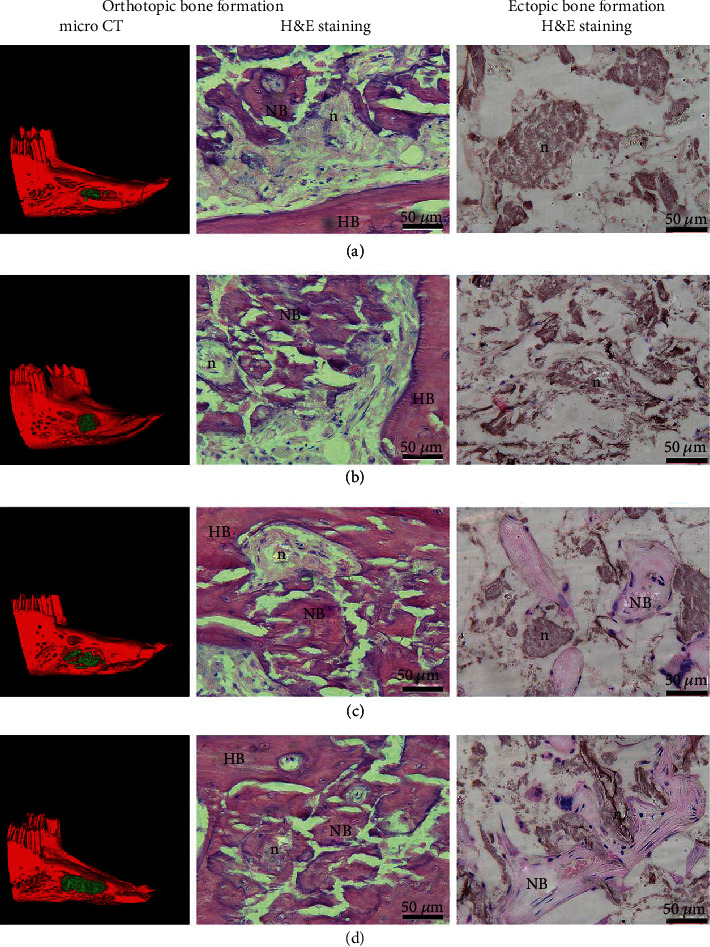
Assessment of engineered bone formation in the *in vivo* ectopic and orthotopic microenvironment after 3 months of surgery. (a) nHAC/PLA. (b) hUC-MSCs+nHAC/PLA. (c) hUC-MSCs+nHAC/PLA+OMD. (d) hUC-MSCs+nHAC/PLA+OMD+rhBMP-7. Scale bar = 50 *μ*m. Green indicated newly formed bone in micro-CT image. HB: host bone; NB: new bone; n: nHAC/PLA.

When the constructs were implanted into jaw bone defects for 3 months, the newly formed mineralized tissue covering the defect could be observed in all grafts by three-dimensional micro-CT reconstruction (Figures 9(a)–9(d)). Hematoxylin and eosin staining showed that the nHAC/PLA (Figure 9(a)), hUC-MSCs+nHAC/PLA (Figure 9(b)), hUC-MSCs+nHAC/PLA+OMD (Figure 9(c)), and hUC-MSCs+nHAC/PLA+OMD+rhBMP-7 (Figure 9(d)) grafts exhibited obvious new bone formation. The new bone edges were arranged in a spindle morphology osteoblasts, and the newly formed bones had new blood vessels passing through.

When the constructs were implanted subcutaneously on the back of SCID mice for 3 months, hematoxylin and eosin staining showed that the nHAC/PLA (Figure 9(a)) and hUC-MSCs+nHAC/PLA (Figure 9(b)) grafts had no bone formation, with a few blood vessels and a large number of residual nHAC/PLA following degradation. The hUC-MSCs+nHAC/PLA+OMD (Figure 9(c)) and hUC-MSCs+nHAC/PLA+OMD+rhBMP-7 (Figure 9(d)) grafts exhibited new bone formation, abundant blood vessels, and active osteoblasts.

**Figure 10:**
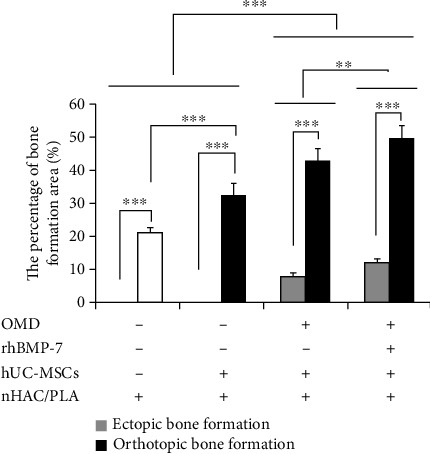
Percentage of bone formation area in the *in vivo* ectopic and orthotopic microenvironment after 3 months of surgery (mean ± SD, *n* = 6). ^∗∗^*P* < 0.01; ^∗∗∗^*P* < 0.001.

The morphometric analysis demonstrated that, in the *in vivo* jaw bone orthotopic microenvironment, the percentage of the bone formation area in the hUC-MSCs+nHAC/PLA (*P* < 0.001), hUC-MSCs+nHAC/PLA+OMD (*P* < 0.001), and hUC-MSCs+nHAC/PLA+OMD+rhBMP-7 (*P* < 0.001) grafts was significantly higher than that in the nHAC/PLA graft. The percentage of bone formation area in the hUC-MSCs+nHAC/PLA+OMD (*P* < 0.001) and the hUC-MSCs+nHAC/PLA+OMD+rhBMP-7 (*P* < 0.001) grafts was significantly higher than that in the hUC-MSCs+nHAC/PLA graft. But the highest percentage of bone formation area was presented in the hUC-MSCs+nHAC/PLA+OMD+rhBMP-7 graft (*P* < 0.01, Figure 10).

In the *in vivo* subcutaneous ectopic microenvironment, the percentage of bone formation area in the hUC-MSCs+nHAC/PLA+OMD and hUC-MSCs+nHAC/PLA+OMD+rhBMP-7 grafts was significantly higher than that in the hUC-MSCs+nHAC/PLA (*P* < 0.001) and nHAC/PLA (*P* < 0.001) grafts. No significant difference was identified between the hUC-MSCs+nHAC/PLA and nHAC/PLA grafts. However, the highest percentage of bone formation area was presented in the hUC-MSCs+nHAC/PLA+OMD+rhBMP-7 graft (*P* < 0.01, Figure 10). Furthermore, the percentage of bone formation area of the same grafts in the *in vivo* jaw bone orthotopic microenvironment was significantly higher than that in the *in vivo* subcutaneous ectopic microenvironment (*P* < 0.001, Figure 10).

## 4. Discussion

The hUC-MSCs are ideal noncontroversial MSC sources in regenerative medicine due to its clinical waste source, a painless collection procedure, faster self-renewal properties, and a high potential for safe cell-based therapies [41]. When used as exogenously transplanted stem cells, the therapeutic efficacy of stem cells is strongly influenced by its surrounding microenvironments [18]. These microenvironments, or niche, provide dynamic physical and chemical cues essential to their survival, proliferation, and differentiation [4]. In recent years, nano- and microengineered scaffolds have been served as a microenvironment to both regulate stem cell fate, increase transplanted cell viability, and improve therapeutic outcomes [42], which can better simulate the microenvironment *in vivo* when combining scaffold and growth factor to promote the directional tissue differentiation [19]. Hence, in this study, the nHAC/PLA, osteogenic media, and rhBMP-7 are hired to investigate the role of the microenvironments in the hUC-MSC-based bone regeneration.

Our isolated cells exhibited typical fibroblastic morphology and self-renewal potential, expressed MSC markers, did not express hematopoietic markers and HLA-DR surface molecules, and possessed multilineage differentiation potential, which suggested that these cells were hUC-MSCs according to the minimal criteria to define as MSCs proposed by the Mesenchymal and Tissue Stem Cell Committee of the International Society for Cellular Therapy [43]. Consistent with other studies [10–12, 15], these cells exhibited osteoblastic phenotypes in the *in vitro* two-dimensional culture microenvironment containing OMD or OMD+rhBMP-7. BMPs have been found to play a good role in promoting differentiation of stem cells to osteogenesis [26–28]. The study showed that BMP-7 enhanced osteogenic differentiation of MSCs derived from elderly osteoporotic bone [19]. The combination of OMD and BMP-7 showed higher osteogenic differentiation of human adipose-derived stem cells than OMD alone [44]. In this study, the higher ALP secretion and mineralized matrix formation suggested that the combination of OMD and rhBMP-7 induced hUC-MSCs to exhibit better osteogenic differentiation ability than OMD alone. This indicated that growth factor rhBMP-7 had a positive effect on osteogenic differentiation of hUC-MSCs in the *in vitro* two-dimensional culture microenvironment.

The basic elements of bone tissue engineering include stem cells, growth factors, and scaffold materials [3]. A scaffold with good biocompatibility can provide a good microenvironment for the proliferation, adhesion, and functional differentiation of cells [20]. The nHAC/PLA is a developed ceramic/polymer composite material and mimics the nano- to microscale hierarchical microstructure of natural, cancellous bones [21]; it has been proved to support stem cells' adhesion, proliferation, and differentiation [22–24]. Similarly, in this study, the nHAC/PLA constructed a three-dimensional culture microenvironment for hUC-MSCs' growth and reproduction. The hUC-MSCs adhered, grew, and proliferated well on the nHAC/PLA. When OMD or OMD+rhBMP-7 was added to this three-dimensional microenvironment, there were changes in the proliferation and differentiation of hUC-MSCs. Cell proliferation progression was shortened; osteogenic differentiation progression was advanced. As cells adapted to these three-dimensional microenvironments (1-3 days), cell proliferation began to be inhibited (5-9 days); the cells began to differentiate into osteoblasts. At day 7 or 14, the genes related to osteogenic differentiation (ALP, OCN, and BMP-2) were significantly upregulated and the corresponding matrix protein secretions were also significantly increased, as determined by RT-PCR, the Roche kits, and ELISA. Our previous study showed that rhBMP-7 alone promoted hUC-MSCs' bone regeneration, though it had no effect on the proliferation of hUC-MSCs seeded on nHAC/PLA during logarithmic growth phase [38]. BMP-7 alone incorporated polycaprolactone scaffold promoting effects on the survival and proliferation rate of the kidney cells [45]. A biphasic, demineralized, and decellularized allograft bone-hydrogel scaffold with a cell-based BMP-7 delivery system promoted cell adhesion, proliferation, and the reconstruction of bone and cartilage defects [46]. In this study, though the *in vitro* three-dimensional culture microenvironment containing OMD or OMD and rhBMP-7 inhibited hUC-MSCs' proliferation, rhBMP-7 attenuated the inhibitory effect of OMD on cell proliferation and enhanced the promoting effect of OMD on osteogenic differentiation of hUC-MSCs. This suggested that growth factor rhBMP-7 had a positive effect on the proliferation and osteogenic differentiation of hUC-MSCs in the *in vitro* three-dimensional culture microenvironment.

The study reported that rhBMP-7 enhanced osteogenic differentiation of murine MSCs and produced a reciprocal expression profile in the expression of endogenous BMPs, as compared with BMP antagonism. Osteogenic differentiation is regulated by a complex network of multiple BMPs that exhibit selective increases and decreases in expression during differentiation [47]. The transient knockdown of BMP-2 using small interfering RNA demonstrated that the osteoinductive properties of BMP-7 are independent of endogenous BMP-2 expression in human MSCs [47]. BMP-7 has been also reported to induce BMP-2 expression in murine MSCs [32]. The mechanism of the effect of rhBMP-7 on the proliferation and osteogenic differentiation of human or animal MSCs is currently controversial, but the fact that osteogenic differentiation is regulated by a complex network of multiple BMPs is fundamentally accepted. In this study, the higher BMP-2 mRNA expression and BMP-2 protein secretion in the *in vitro* three-dimensional culture microenvironment containing OMD and rhBMP-7 indicated that the endogenously produced BMPs might serve an important regulatory role in the osteogenic differentiation of hUC-MSCs.

To reconstruct bone tissue locally *in vitro* and *in vivo*, at least, stem cells activities, growth factors regulating cellular activities, matrices produced by stem cells, scaffold providing the microenvironment, mineral ions in the microenvironment, and the mineralization process must be taken into consideration [48]. Bone is a composite structure composed of nanosized inorganic hydroxyapatite (HAp) and organic components composed mainly of fibres of the structural protein type I collagen, as well as other matrix proteins [49]. In the process of bone formation, the continuous deposition of hydroxyapatite in the extracellular matrix for mineralization requires appropriate Ca^2+^ and PO_4_^3-^ concentrations outside the extracellular matrix [50]. In this study, nHAC/PLA is composed of hydroxyapatite, collagen, and polylactic acid, and the content of hydroxyapatite is 45% ± 5%. When nHAC/PLA alone was cultured in serum-free medium for 14 days, the concentrations of Ca^2+^ and PO^3-^_4_ in the microenvironment were 1.5817 mmol/l and 1.0683 mmol/l, respectively. Then, the ion concentration at the interface of the nHAC/PLA and the ion concentration in the serum-free medium maintained a balance. When the hUC-MSCs were seeded on the nHAC/PLA and cultured in serum-free medium for 14 days, the cell adhesion, growth, proliferation, and differentiation caused the nHAC/PLA degradation to break the ion balance, leading to an increase in the concentration of Ca^2+^ and PO^3-^_4_ in the microenvironment. When OMD or OMD+rhBMP-7 was added into this microenvironment for 14 days, the expressions of genes related to osteogenic differentiation (ALP, OCN, and BMP-2) were significantly upregulated, and the corresponding matrix protein secretions were significantly increased. At the same time, the concentration of Ca^2+^ and PO_4_^3-^ in the microenvironment significantly increased; it resulted in the continuous deposition of hydroxyapatite in the extracellular matrix for more mineralization as determined by Alizarin red staining and quantitative analysis, while rhBMP-7 significantly enhanced the promoting effect of OMD on Ca^2+^ and PO_4_^3-^ concentration as well as mineralized matrix formation. The studies confirmed that bioactive ceramic coatings depend on its interfacial dissolution, precipitation, and ion exchange reactions to affect cellular proliferation, differentiation, collagen deposition, and mineralization [51]. In this study, we found that the nHAC/PLA, hUC-MSCs, OMD, and rhBMP-7 interacted and influenced each other. OMD or OMD+rhBMP-7 affected the adhesion, proliferation, and differentiation of hUC-MSCs. The adhesion, proliferation, and differentiation of hUC-MSCs affected the degradation of nHAC/PLA. Ca^2+^ and PO_3_^−4^ ions derived from the nHAC/PLA and OMD affected the gene expression as well as protein secretion related to osteogenic differentiation of hUC-MSCs. These factors and their formed microenvironments played a key role in the mineralization process of hUC-MSCs. While the *in vitro* three-dimensional culture microenvironment engineered by hUC-MSCs, nHAC/PLA, OMD, and rhBMP-7 led to more successful cell proliferation, differentiation, and mineralization for the *in vitro* tissue-engineered bone, and in this *in vitro* three-dimensional culture microenvironment, hU-CMSCs exhibited a normal diploid karyotype.

*In vivo* studies on the comparison of the bone formation ability of hUC-MSCs in ectopic and orthotopic microenvironments are rare, to the best of our knowledge. Of note, when the sterile, resorbable 10 × 4 × 3 mm nHAC/PLA scaffolds with/without 1 × 10^7^ cell/cm^2^ cultured *in vitro* for 7 days were transplanted subcutaneously into the back of SCID mice for ectopic bone formation and transplanted into rabbit jaw bone defect for orthotopic bone formation, both nHAC/PLA and hUC-MSCs+nHAC/PLA exhibited bone formation in orthotopic microenvironment, but not in ectopic microenvironment. This suggested that the interaction of scaffolds, stem cells, and i*n vivo* transplanted microenvironment played an important role in bone regeneration. The studies verified that the pore size of scaffolds needed for bone ingrowth is ≥100 *μ*m, and the most favorable pore size for new bone formation is 300-400 *μ*m [48]. In this study, the porosities of nHAC/PLA were 70-88%, and the pore sizes were 300 ± 250 *μ*m; the favorable interconnected pores promoted the diffusion of oxygen and nutrients into nHAC/PLA in orthotopic microenvironment and made native host cells migrate into materials and proliferate, differentiate, produce blood vessels, and finally, form new bone within the porous structures. The recruited native host cells from orthotopic microenvironment may be responsible for the subsequent bone formation and remodeling. Therefore, orthotopic microenvironment served an important role in the bone formation of nHAC/PLA itself. Furthermore, the hUC-MSCs+nHAC/PLA exhibited a significantly higher percentage of bone formation area compared with the nHAC/PLA in orthotopic microenvironment. This suggested that hUC-MSCs transplanted into the orthotopic microenvironment served a role in bone regeneration. The studies reported that hUC-MSCs, human-induced pluripotent stem cells, and hBM-MSCs exhibit a significantly larger amount of new bone compared with a cell-free macroporous calcium phosphate cement control in rat cranial defects [49]. These results suggested that an *in vivo* orthotopic microenvironment provides signals to promote *in vitro* uninduced MSCs differentiate into osteoblasts to participate in the process of bone regeneration. Both the hUC-MSCs+nHAC/PLA+OMD and hUC-MSCs+nHAC/PLA+OMD+rhBMP-7 exhibited bone formation in ectopic and orthotopic microenvironments, the higher bone formations were presented in hUC-MSCs+nHAC/PLA+OMD+rhBMP-7 and orthotopic microenvironment. This demonstrated that *in vitro* three-dimensional culture microenvironment played an important role in stem cell-based bone regeneration. The exogenously added rhBMP-7 enhanced bone formation ability of hUC-MSCs. The results indicated that the combination of *in vitro* hUC-MSCs+nHAC/PLA+OMD+rhBMP-7 microenvironment and *in vivo* orthotopic microenvironment provided a more optimized niche for bone regeneration of hUC-MSCs.

## 5. Conclusion

In summary, our results showed that the *in vitro* three-dimensional culture microenvironment formed by the regenerative engineered bone and the *in vivo* orthotopic microenvironment closely matched that of the bone tissue in its native state may be essential for sufficient and timely bone regeneration. This study elucidates that hUC-MSCs and their local microenvironment, or niche, play an important role in hUC-MSC-based bone regeneration. The endogenously produced BMP may serve an important regulatory role in the process.

## Data Availability

The data used to support the findings of this study are available from the corresponding author upon request.
